# Knowledge and practices of seeking informed consent for medical examinations and procedures by health workers in the Democratic Republic of Congo

**DOI:** 10.4314/ahs.v21i1.58

**Published:** 2021-03

**Authors:** Doudou Nzaumvila, Patrick Ntotolo, Indiran Govender, Philip lukanu, JD Landu Niati, Didier Sanduku, Tombo Bongongo

**Affiliations:** 1 Sefako Makgotho Health Sciences University; 2 Universite Protestante du Congo; 3 University of Pretoria, Department of Family Medicine; 4 Sanru

**Keywords:** Informed consent, medical examinations, procedures by health workers in the Democratic Republic of Congo

## Abstract

**Background:**

Informed consent (IC) is linked to the ethical principle of respecting patient autonomy, respect for human rights and ethical practice, while in many countries it is a standard procedure. Anecdotally, it should be noted that in the Democratic Republic of Congo (DRC) in many instances ICs are not obtained systematically. To date, no research appears to have been conducted on this matter. This study aimed to assess the knowledge and practice of obtaining IC from patients among health care providers (HCP) in the DRC.

**Methods:**

This was a cross-sectional study, with a convenient sampling of 422 participants. Data from the questions were collected on an imported Microsoft Excel spreadsheet for review at INSTAT.TM The authors set IC's accurate knowledge and practice at 80% or higher. The Fisher Exact test was used to compare categorical association results, and a p-value < 0.05 was considered statistically significant.

**Results:**

Results showed that giving information in detail to patients on their medical condition was associated with formal training on medical ethics and IC (p: 0.0028; OR: 1.894; CI: 1.246 to 2.881), which was also associated with answering the patient's questions in detail (p: 0.0035; OR: 1.852; CI: 1.236 to 2.774). About 127(30.09 %) of participants scored 80% or higher. Extracurricular training was associated with withholding information from patients, up to 27 times more than other factors (p< 0.0001; OR: 27.042; CI: 13.628 to 53.657). when it comes to get IC, HCP with many years of practice scored better than others, in one of the question the odd ratio was closer to 7 (p< 0.0001; OR: 6.713; CI: 4.352 to 10.356). Only 47(11.14%) of the participants scored 80% or more of the questions about practice of IC.

**Conclusion:**

For a variety of reasons, knowledge and practice of IC among HCPs was very low. A common programme for the country as part of formal training might lead to an improvement. In addition, patients' education on IC should be displayed in waiting areas at all medical centres.

## Introduction

Informed consent is the process whereby an health care providers (HCP) discloses appropriate information to a competent patient in order for the patient to make a voluntary decision as to whether to accept or refuse treatment.^1^ This process is integral to a patient's autonomy and enables him or her (or a surrogate) to participate in medical decision-making. With the exception of emergencies, it is currently accepted as an ethical obligation by many health professional governing bodies for any HCP, before a medical examination and/or surgical procedure takes place, to obtain informed consent (IC) from the competent patient or surrogate. In other words, IC is closely linked to the ethical principle of respecting patient autonomy, respect of human rights and ethical practice.^2^,^3^ Furthermore, the constitutions of many countries today, at different levels, protect and guarantee universal basic human rights. That makes IC a legal requirement for any HCP before any medical examination or procedure takes place.^1^,^2^ IC is therefore a professional and ethical issue emanating from the fiduciary responsibility of the HCP towards his/her patient.^3^,^4^

The current standard requires the following five elements for IC to be valid: (i) competence; (ii) voluntariness; (iii) information disclosure; (iv) understanding of information disclosed; and (v) authorisation or consent to the medical procedure or treatment.^5^ The United States of America was the first to introduce the ethical and legal obligation for IC, which dates back to the 1960s, followed by European countries almost ten years later.^6^–^8^ More recently, in many other countries, under the direction of international organisations and local human rights activists, IC has been made a standard procedure, although with some difficulties in implementation due to local cultures and beliefs.^2^,^9^–^11^

African countries face two different challenges concerning IC: its implementation and its practice^11^. The implementation of IC faces several barriers. Among them is patient illiteracy and patriarchal attitudes, whereby a person cannot give consent without the prior consent of a third party such as a husband, community leader or elder, due to their important social role.^11^ A further barrier is medical paternalism, whereby with the best of his/her knowledge, and without any intention to harm, the HCP decides what is best for the patient.^12^ Although one could agree that this respects the principle of beneficence, it clearly offends patient autonomy. The practice of IC is challenged by the situation of multicultural societies like South Africa.^13^ Other factors were also reported such as multilingualism, poverty, education, unfamiliarity with libertarian rights based on autonomy, and power asymmetry between doctors and patients in many African countries.^13^–^15^

Since colonial times until about the 1980s, the health system in the DRC was strictly regulated by the Health Code of March 19, 1952.^16^ Since achieving independence in 1960, the DRC has honoured a number of international commitments, such as the 1948 Universal Declaration of Human Rights, accession to the World Health Organization (WHO), and many others aimed at promoting patients' autonomy. Anecdotally, it has been reported that in many instances, IC is not obtained systematically as requested from an HCP. This brings unnecessary complaints and dissatisfaction from patients and relatives especially if problems arise. Even though this may be done without a premeditated will to do harm, it unfortunately denies patients the autonomy to make choices regarding their medical care, without attempts by HCPs trying to influence this decision.^2^ To date, except for literature on consent in medical research in the DRC, 17 no research appears to have been conducted on IC for medical examinations or procedures in that country. Consequently, the current study aimed to assess knowledge of IC and to evaluate the practice of obtaining IC among health workers in the DRC.

## Methods

The study design was a cross-sectional, with a convenient sampling of 422 participants. The study population comprised HCPs who are involved in patient care in different positions, namely: doctors (interns, medical officers, registrars and specialists); nurses (all categories: registered nurses, professional nurses); other medical professionals such as physiotherapists, psychologists, radiographers were grouped as healthcare science professionals (HSPs); students (all categories: medical students, student nurses, HSPs students).

The study was set in three provinces of the DRC: (i) Kongo Central Province (CH Lamba, CM Christ-vie, CM La Famille, HGR IME, Hospital of Nsona Nkula); (ii) North Kivu Province (Heal Africa Hospital); (iii) Kwilu Province (HGR Kikwit Sud, HGR Kikwit Nord). Data collection was done by the means of a piloted, structured and self-administered questionnaire adapted from a previous study.4 The questionnaire was modified by the researcher based on the aim and objectives of the current study. It was piloted in two hospitals which are not part of this study. This helped to adjust some of the questions. This adjusted questionnaire was reviewed by two consultants in Family Medicine (as peer review) and by a senior in research (as expert) at the Department of Family Medicine/ SMU before it can be used in DRC's hospitals. The questionnaire was written in English and translated in French, which is the spoken language in DRC. The translation was done by the researcher and the two consultants who are bilingual. Each and every questionnaire was marked by a number (e.g. 1,2,3,4, etc.). It contained questions related to different variables such as socio-demographics (age, gender, marital status, profession or qualifications: doctor, nurse, physiotherapist, student, location, years of experience, IC: knowledge, practice.

There were three trained medical officers who were introducing the study (aim and objectives) to the HCPs in each province. Questionnaires were handed over to the HCPs who consented to participate into the study. A period of 25–30 minutes was allowed for participants to complete the questionnaire. Variables in the questionnaire included age, gender, profession (doctor, nurse, phlebotomist, physiotherapist, student, etc.), and years of experience. They returned them to the trained medical officers after they have completed. No remuneration was attached to the completion of questionnaire. Data emanating from the questions was captured on a Microsoft Excel spreadsheet imported to INSTATTM for analysis. The authors set the accurate knowledge and practice of IC at greater or equal to 80%. We used the Fisher Exact test to compare categorical data for association, and a p-value < 0.05 was considered statistically significant.

### Ethical considerations

Permission to use and modify the questionnaire was obtained from authors who did similar studies, permission was obtained from the relevant authorities of different health care facilities where the study was conducted and we obtained ethical clearance to conduct this study from the ethics committee SREC and SMUMREC of the Sefako Makgatho Health Sciences University (SMUMREC/M/128/2018:IR). Written consent was also required from participants.

## Results

### Demographic characteristics

It was shown that 40.76% of participants were between 30 and 39 years of age; the youngest was 18 and the oldest was 73. The mean standard deviation was 38.12 (±0.49) years. There were more male 239(56.64%) than female participants. Married participants accounted for 217(51.42%). Of the participants 150(35.55%) had between 15 and 19 years of years of practice and nurses represented 267(63.27%) of the study sample, as indicated in Table I.

**Table I:** Baseline characteristics

Characteristics	n 422	Percentage (%)
Age (years)		
18–29	84	19.90
30–39	172	40.76
40–49	105	24.89
50–59	43	10.19
60–69	17	4.03
≥ 70	1	0.24

Gender		
Female	183	43.36
Male	239	56.64

Marital status		
Divorcees	13	3.08
Living together	18	4.27
Married	217	51.42
Separated	7	1.66
Single	149	35.31
Widower	18	4.27

Occupation		
Dentists	7	1.66
HSPs	55	13.03
Medical officers	56	13.27
Medical specialists	15	3.55
Nurses	267	63.27
Students	22	5.21

Years of practice		
0–4	133	31.52
5–9	21	4.98
10–14	55	13.03
15–19	150	35.55
20–24	23	5.45
25 and more	40	9.48

### Knowledge of IC

It transpired that 250(59.24%) had been thorough with regard to IC regulations during their formal training with a higher percentage found among medical specialists (14; 94%). We also noted that 213(50.48%) of HCPs, especially students, did not know that the procedure of obtaining IC for treatment is regulated by law (18; 81.81%). Results showed that 296(70.14%) agreed that obtaining an IC is an ethical obligation with 100% of medical specialists (doctors) agreeing. However, 217(51.44%) disagreed that it is a legal obligation, a result particularly prevalent among HSPs professionals (33; 60%).

The majority of participants (356; 84.36%) were not of the opinion that an HCP may be allowed to deliberately withhold the right of information from the patient, mainly from the HSPs group (51; 92.73%). A total of 55% of those of the opposite opinion stated that they could withhold information if the patient was not competent (Table II).

**Table II:** Knowledge of IC

**Have you ever been trained to** **medical ethics especially about** **IC?**	**Yes, during my** **training n(%)** **250(59.24)**	**No** **n(%)** **172(40.76)**	**p-value**
Dentists HSPs Medical officers Medical specialists Nurses Students	6(85.71) 28(50.91) 41(73.21) 14(93.33) 145(54.31) 16(72.73)	1(14.29) 27(49.09) 15(26.79) 1(6.67) 122(45.69) 6(27.27)	0.2487 0.1879 0.0279* 0.0059* 0.0076* 0.2650
**Does the law regulate the procedure of** **obtaining IC for treatment?**	**Yes** **n(%)** **209(49.52)**	**No** **n(%)** **213(50.48)**	**p-value**
HSPs Dentists Medical officers Medical specialists Nurses Students	21(38.18) 2(28.5) 29(51,79) 13(86.67) 140(52.43) 4(18.18)	34(61.82) 27(48.21) 2(13.33) 127(47.57) 18(81.81)	0.4447 0.7748 0.0033* 0.1301 0.0035*
**Is obtaining an IC an ethical obligation?**	**Yes** **n(%)** **296(70.14)**	**No** **n(%)** **126(29.86)**	**p-value**
HSPs Dentists Medical officers Medical specialists Nurses Students	37(67.27) 6(85.71) 48(82.27) 15(100) 178(66.67) 12(54.54)	18(32.73) 1(14.29) 8(17.73) 0 89(33.33) 10(45.46)	0.6370 0.6796 0.0071* 0.0074* 0.0470 0.1482
**Is obtaining an IC a legal obligation?**	**Yes** **n(%)** **205(48.58)**	**No** **n(%)** **217(51.42)**	**p-value**
HSPs Dentists Medical officers Medical specialists Nurses Students	22(40) 5(71.42) 35(62.5) 7(46.66) 126(47.19) 10(45.45)	33(60) 2(28.58) 21(37.5) 0 141(52.81) 12(54.55)	0.1942 0.2726 0.0309* 0.0061* 0.4803 0.8289
**Can an HCP deliberately withhold the right of** **information from a patient?**	**Yes** **n(%)** **66(15.64)**	**No** **n(%)** **356(84.36)**	**p-value**
HSPs Dentists Medical officers Medical specialists Nurses Students	4(7.27) 1(14.29) 8(14.29) 4(26.27) 47(17.60) 2(9.09)	51(92.73) 6(85.71) 48(85.71) 11(73.73) 220(82.40) 20(90.91)	0.0739 1.0000 0.8461 0.2687 0.1654 0.5515
**Who should educate the patient on the IC?**	**Healthcare worker** **n(%)** **340(80.57)**	**Others** **n(%)** **82(19.43)**	**p-value**
HSPs Dentists Medical officers Medical specialists Nurses Students	47(84.45) 6(85.71) 50(89.29) 13(86.67) 208(77.90) 16(72.73)	8(15.55) 1(14.29) 6(10.71) 2(13.33) 59(22.1) 6(27.27)	0.3672 1.0000 0.1011 0.7456 0.0748 0.4033

Most of participants (350;82.94%) assumed that because a patient requests a consultation he/she agrees to an examination.

The authors set the accurate knowledge of IC at greater or equal to 80%, and noted that figure in 127(30.09%) of participants. The medical specialist category was the only one to have more than 50% of its members who scored 80% or above (Figure I).

**Figure I:**
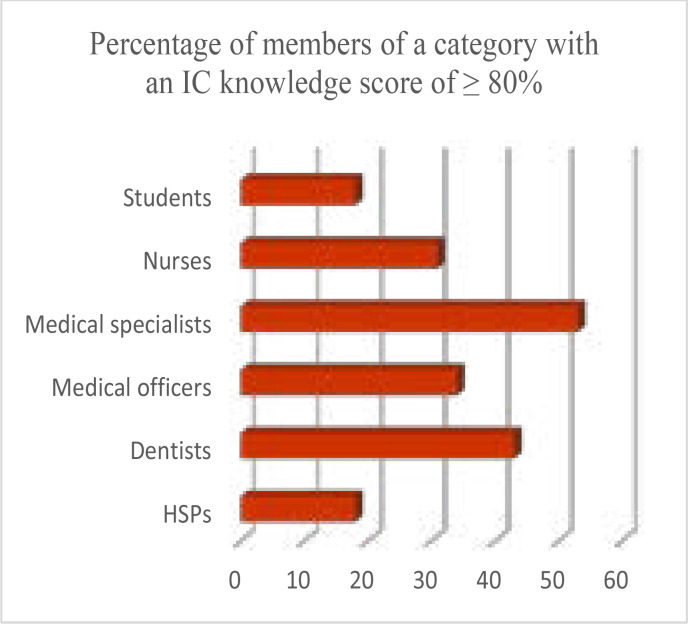
Accurate knowledge of IC

### Practice of obtaining IC by health care workers

When it comes to who should educate patients, it was noted that 360(80.57%) agreed that it was the duty of the HCP with 50(89.29%) medical doctors making this statement. However, only 192(45.50%) admitted always obtaining consent before an examination, and 207(49.50%) before any procedure, with the exception of dentists who scored differently from the last two groups, i.e. 6(85.71%). Regarding mutual agreement on the method of treatment, it was reported by HCPs as 137(34.42%) with a different score, 12(85.71%), than the majority for medical specialists.

The explanation to patients was always given in detail by 151(35.78%) participants as well as responses to patients' questions also being answered in detail. In the case of admission, information to patients about the possible length of their hospital stay is given in 73(17.30%) of cases. (Table III). The existence of a consent form for any medical or surgical act in the health care facilities was confirmed by 70(16.59%) HCPs.

**Table III:** Practice of obtaining IC by health care workers

I ask the patient for permission to examine him/her	Always n(%) 192(45.50)	Not always n(%) 230(54.50)	p-value
Allied Dentists Medical officers Medical specialists Nurses Students	28(50.91) 6(85.71) 23(41.07) 10(66.67) 110(41.20) 15(68.18)	27(9.09) 1(14.29) 33(59.93) 5(33.33) 127(49.80) 7(31.82)	0.3888 0.0504 0.5647 0.1155 0.6942 0.0455*
I ask the patient for permission to perform a procedure	Always n(%) 207(49.50)	Not always n(%) 215(50.50)	p-value
Allied Dentists Medical officers Medical specialists Nurses Students	26(42.27) 6(85.71) 29(51.79) 10(66.67) 121(45.32) 15(68.18)	29(32.73) 1(14.29) 27(17.73) 5(33.33) 146(54.78) 7(31.82)	0.8851 0.0635 0.6698 0.1947 0.0548 0.0801
I inform patients about their medical condition and treatment procedures	Always in detail n(%) 151(35.78)	Not always n(%) 271(64.22)	p-value
Allied Dentists Medical officers Medical specialists Nurses Students	21(38.18) 5(71.43) 26(46.43) 11(73.33) 73(27.43) 15(68.18)	34(61.82) 2(28.57) 30(37.57) 4(26.67) 194(52.81) 7(54.55)	0.7632 0.1032 0.0988 0.0041* < 0.0001* 0.0022*
I answer the patient's questions	Always in detail n(%) 174(41.23)	Not always n(%) 248(58.77)	p-value
Allied Dentists Medical officers Medical specialists Nurses Students	21(38.18) 4(57.14) 35(62.5) 11(73.73) 82(30.71) 16(72.73)	34(61.73) 3(43.85) 21(37.5) 4(26.27) 220(69.29) 6(27.27)	0.6618 0.4537 0.0007* 0.0144* <0.0001* 0.0031*
Patients usually choose the treatment method	Mutual agreement n(%) 137(34.42)	Suggested by me n(%) 261(65.58)	p-value
Allied Dentists Medical officers Medical specialists Nurses Students	22(45.89) 3(50) 21(48.48) 12(85.71) 74(29.13) 5(23.91)	26(54.17) 3(50) 34(51.52) 2(14.29) 180(70.87) 16(76.19)	0.1042 0.4185 0.0946 <0.0001* 0.0042* 0.3522
In case of admission, do you inform patients of the possible length of their hospital stay?	Always n(%) 73(17.30)	Not always n(%) 349(71.09)	p-value
Allied Dentists Medical officers Medical specialists Nurses Students	11(20) 0 11(19.64) 4(26.67) 45(16.85) 2(9.09)	44(80) 7(100) 45(90.36) 11(73.33) 222(83.15) 20(90.91)	0.5681 0.6099 0.5748 0.3057 0.7900 0.3950

A score of 80% and more was considered as accurate for practice of IC; what was noted is 47(11.14%) by medical specialists. None of the health care categories had at least 50% of members who scored 80% or more, as is evident from Figure 2.

**Figure II:**
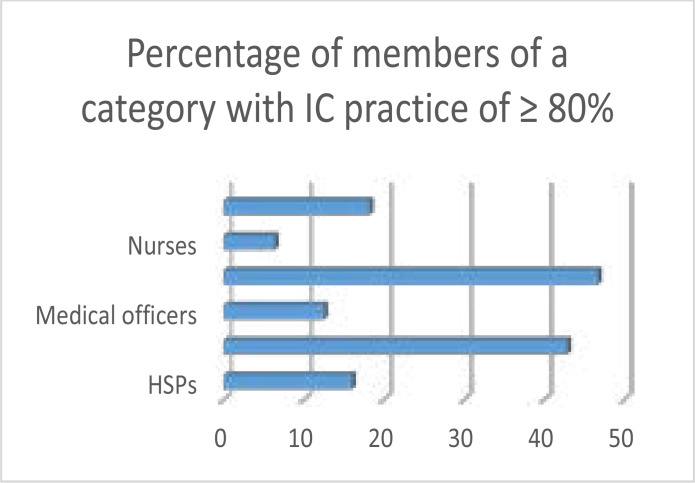
Accuracy of practice of IC

### Bivariate analysis

The authors considered four factors that can be associated with the knowledge and practice of IC. Extracurricular training (any teaching that is not part of a structured university program) was associated with withholding information from the patients, up to 27 times more than the others (p< 0.0001; OR: 27.042; CI: 13.628 to 53.657). Years of practice was associated with three of the five questions on knowledge of IC, with an almost seven times possibility of a good answer to the question about who should educate patients on IC (p< 0.0001; OR: 6.713; CI: 4.352 to 10.356). Table IV displays more associations between knowledge of IC and the four factors mentioned above.

**Table IV:** Bivariate analysis of knowledge of IC

Knowledge of health workers on IC	p-value	Odd ratio (95% confidence interval)
**Does the law regulate the procedure of** **obtaining IC for treatment?**		
Age	0.0080*	0.5839(0.3960 to 0.8611)
Formal training on ethics and IC	< 0.0001*	3.267(2.173 to 4.914)
Extra-curricular training on IC	0.8966	0.9449(0.5673 to 1.574)
Years of practice	0.5587	1.140(0.7779 to 1.671)

**Is obtaining an IC an ethical obligation?**		
Age	0.2381	0.7589(0.4961 to 1.161)
Formal training on ethics and IC	< 0.0001*	3.167(2.056 to 4.878)
Extracurricular training on IC	1.0000	1.030(0.5893–1.801)
Years of practice	< 0.0001*	0.4000(0.2554 to 0.6266)

**Is obtaining an IC a legal obligation?**		
Age	0.6244	0.9040(0.6151 to 1.329)
Formal training on ethics and IC	0.0230	1.579(1.067 to 2.336)
Extracurricular training on IC	0.7747	1.111(0.6388 to 1.933)
Years of practice	0.6986	1.108(0.6649 to 1.845)

**Can an HCP deliberately withhold the right** **of information from the patient?**		
Age	0.6244	0.5123(0.6151 to 1.329)
During formal training on ethics and IC	0.5873	0.8566(0.5040 to 1.456)
Extracurricular training on IC	< 0.0001*	27.042(13.628 to 53.657)
Years of practice	< 0.0001*	0.06176(0.03396 to 0.1123)

**Who should educate the patient on the** **issue** **of providing consent for treatment?**		
Age	0.0149*	0.5722 (0.3009 to 0.8721)
During formal training on ethics and IC	0.0010*	2.310(1.415 to 3.771)
Extracurricular training on IC	0.5107	0.7961(0.4291 to 1.477)
Years of practice	< 0.0001*	6.713(4.352 to 10.356)

When it comes to the practice of IC, results showed that giving information in detail about their medical conditions was associated with formal training on medical ethics and IC (p: 0.0028; OR: 1.894; CI: 1.246 to 2.881), which was also associated with answering the patient's questions in detail (p: 0.0035; OR: 1.852; CI: 1.236 to 2.774). Extracurricular training on IC was associated with mutual agreement on the choice of treatment (p< 0.0001; OR: 13.277; CI: 6.329 to 27.852). The remaining details on bivariate analysis are in Table V.

**Table V:** Practice of obtaining IC

Practice of obtaining IC	p-value	Odd ratio (95% CI)
**I ask the patient for permission to examine him/her**		
Age	1.000	0.9890(0.6720 to 1.456)
Formal training on ethics and IC	0.0104*	1.763(1.144 to 2.633)
Extracurricular training on IC	0.0027*	0.4399(0.2565 to 0.7545)
Years of practice	0.84456	1.050(0.7142 to 1.543)

**I ask permission to before I perform a procedure**		
Age	0.3571	2.164(0.8189 to 1.836)
Formal training on ethics and IC	0.4292	0.8471(0.5741 to 1.250)
Extracurricular training on IC	0.0002*	1.226(1.455 to 3.220)
Years of practice	0.4854	1.167(0.7883 to 1.727)

**I inform patients about their medical condition and** **treatment procedures in details**		
Age	< 0.0001*	0.3220(0.2083 to 0.4975)
Formal training on ethics and IC	0.0028*	1.894(1.246 to 2.881)
Extracurricular training on IC	0.4991	1.207(0.7146 to 2.039)
Years of practice	0.5918	1.149(0.6804 to 1.939)

**I answer the patient's questions in detail**		
Age	0.4854	1.167(0.7883 to 1.727)
Formal training on ethics and IC during	0.0035*	1.852 (1.236 to 2.774)
Extracurricular training on IC	0.5982	0.8516(0.5042 to 1.438)
Years of practice	0.60499	0.8646(0.5210 to 1.435)

**Patients usually choose the treatment method in** **mutual** **agreement**		
Age	< 0.0001*	0.05138(0.03109 to 0.08491)
Formal training on ethics and IC	0.2912	0.7948(0.5261 to 1.201)
Extracurricular training on IC	< 0.0001*	13.277(6.329 to 27.852)
Years of practice	0.0199*	0.6123(0.4102 to 0.9140)

**In case of admission, do you inform patients about** **the** **possible length of their hospital stay?**		
Age	0.6049	0.8646(0.5210 to 1.435)
Formal training on ethics and IC	0.0513	1.728(1.015 to 2.942)
Extracurricular training on IC	0.1695	0.5587(0.2552 to 1.223)
Years of practice	0.6996	0.8768(0.5288 to 1.454)

The comparison between knowledge and practice showed a significant difference in the professional category of nurses (Figure III).

**Figure III:**
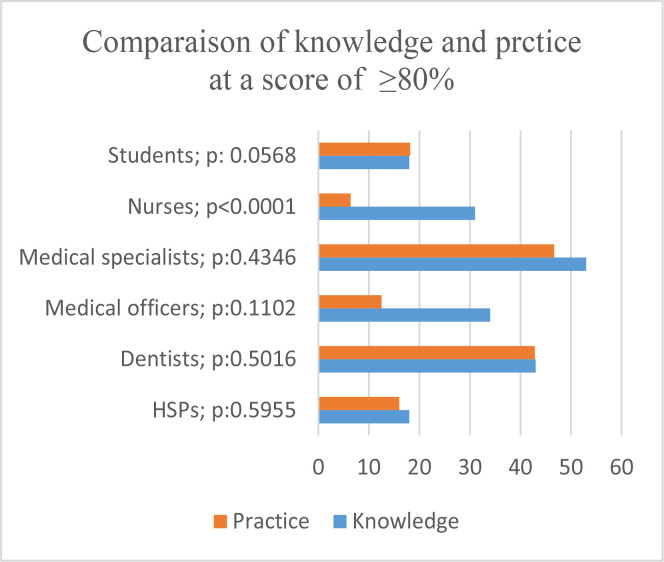
Comparison of knowledge and practice at ≥80%

At ≥80% of knowledge and practice, were among the four factors identified to have a possible influence. The results showed that HCPs with formal training had four times the odds of possessing accurate knowledge; those with many years of practice had six times the odds of good practice at ≥ 80% (Table VI).

**Table VI:** Bivariate analysis of knowledge and practice of IC at ≥ 80%

Characteristics	n(%)	p-value	OR(CI at 95%)
Knowledge of IC at ≥80%	127(30.09)		

Age	53(41.73)	<0.0001*	0.4259(0.2786 to 0.6510)
Formal training on medical ethics and	102(80.31) 22(17.32)	<0.0001*	4.052(2.474 to 6.638)
IC Extracurricular training	52(40.95)	0.2920	1.375(0.7778 to 2.432)
Years of practice		0.0261*	0.6178(0.4054 to 0.9414)
	47(11.14)		
Practice of IC at ≥80%			
	27(57.45)		
Age	41(87.23)	<0.0001*	1.049(0.5682 to 1.938)
Formal training on medical ethics and IC	6(13.77)	<0.0001*	5.527(2.249 to 13.096)
Extracurricular training	23(48.94)	0.5375	0.6979(0.2844 to 1.713)
Years of practice		<0.0001*	6.529(3.417 to 12.473)

The comparison between knowledge and practice within the categories of HCs highlighted a significant difference for nurses with a p-value < 0.0001.

## Discussion

Our study showed that among HCPs there are slightly more males than females (Table I), statistically in keeping with other studies done among HCPs.4,18,19 However, other studies have presented another figure of distribution between males and females.^3^,^20^ This difference related to the type of study done by researchers. This seems to rely on HCP's willingness to participate in this study instead of a survey distribution of healthcare workers.

In our series, the great proportion of medical specialists (93%) admitted having had formal training on medical ethics and IC, in contrast with the remaining participants of the sample. There were associations between health care workers with formal training in medical ethics and IC and who answered in the affirmative that the law regulates IC; it is an ethical obligation, and lastly that it is the duty of the health care worker to educate patients on this matter. Analysis of the odds ratio indicated that there was more than three times the possibility of a positive answer to the first two questions.

Furthermore, at 80% and more of knowledge on IC, medical specialists were at the top in this study with 53% while the next health care category (dentists) was at 10% below. It was also pointed out that formal training in medical ethics and IC had an association with knowledge at ≥ 80%, four times more than others as an odds ratio. Jukić and colleagues4 studied the knowledge on IC for medical procedures of specialists from six Croatian hospitals and also pointed out the crucial part played by formal training with regard to IC. Empowering students with knowledge will without doubt assist them in future and it is essential that these students be educated on the topic.^21^–^23^

Our results established that 81.81% of students ignored the fact that the law regulates the procedure of obtaining IC, in comparison with the figure of 50.48% of HCPs in general. However, 70.14% did agree that obtaining an IC is an ethical obligation. This is an alarming situation as many health care workers in other parts of the word have been legally charged.^24^ In this regard the findings of Jukić and colleagues contrasted with ours. This is most probably due to the paternalistic attitude among patients and health care workers in the DRC where there are very few lawsuits, thereby reducing the visible involvement of the law^17^. On the one hand, what opens the door to inadequate practice of IC can be attributed to poor knowledge, and lack of familiarity with the ethical rules in place.^4^ On the other hand, in medical schools in the DRC medical ethics is a subject taught for only a few hours.4 It is to be expected that theoretical knowledge could, and should, contribute to the informed process and practice.^25^ In many countries, where there is a likelihood of claims in the instance of complications, health care workers are intensively trained to gain approval before medical examinations and procedures,^4^,^26^ a practice not widely observed in the DRC.

Although the majority of HCPs agreed that it was their responsibility to educate patients on IC, less than 50% (34.42%) confirmed that they always obtained consent prior to an examination or to any procedure as well as mutual agreement for the method of treatment. This was at greater or equal to 80% of practice of IC(11.14%). This is without animus nocendi, i.e. a clear indication of human rights' violation by the majority of health care workers and a serious issue where legal protection had been withheld from patients. After independence, 59 years ago, the DRC committed itself to the Universal Declaration of Human Rights, which motivates patients' autonomy; therefore IC is obligatory. Yet to date there has been no clear advocacy for patients' basic right to protection. This problem is not endemic in the DRC; countries in other parts of the globe experience serious issues with IC.^4^,^27^ The findings here are in keeping with the setting and situation noted in some African countries.^10^,^11^

We conducted this study mainly in rural areas where quite possibly several barriers such as illiteracy and patriarchal attitudes exist. It was noted that due to illiteracy and without the approval of a third party such as a husband, group official or teacher, some patients might not grant permission for a procedure because of cultural constraints.^11^ The authors argue that this could discourage HCPs from actively educating and obtaining an IC from a patient but instead acts in good faith. Such a stance promotes medical paternalism, which will require strong clinical, legal, and policy commitment from the country's medical authorities for positive sustainable change. The health worker chooses what is appropriate for the individual with the greatest of his understanding and without any purpose to hurt.^12^ Multiculturalism, multilingualism, poverty, education, unfamiliarity with libertarian rights based on autonomy, and power asymmetry between doctors and patients in many African countries^13^,^14^,^15^ could also have played a role in the setting where this research was based.

## Limitations

The authors recognized some limitations to this study, the first being the methods of sampling and the collection of data. Due to connectivity problems (post office and internet access), data could only be obtained from local health care facilities, or where authors work; The second limitation was the settings. We conducted this study in most rural areas where, as in urban areas, paternalism can be expected. Finally, there were some closed ended questions or structured questionnaires where respondents were not permitted to express their views or feelings other than the way they were in the questionnaire.

## Conclusion

For a variety of factors the knowledge and practice of obtaining IC among HPCs in this study was relatively low. Medical specialists score better than other HCPs and students. Though the DRC pledged to the Universal Declaration of Human Rights, at the moment we conducted this study, there was no strong support for the fundamental right to protection of patients. Although HCP paternalism is not a reciprocal intent to harm, the current figure may be modified by an attempt by their positive involvement.

## Recommendations

It would be appropriate for the government of the DRC to generate a common programme as part of medical training across the whole country. It should be presented on the various teaching levels with both formative and summative components. In addition, full information on IC should be displayed in all waiting areas at clinics and hospitals.

Key factors to improve the current figures include thorough formal training on IC during an HCP's professional studies and a powerful and sustainable advocacy for patients' fundamental right to protection by these health care workers when they engage with patients.
